# Structures of permuted halves of a modern ribose-binding protein

**DOI:** 10.1107/S205979832201186X

**Published:** 2023-01-01

**Authors:** Florian Michel, Sooruban Shanmugaratnam, Sergio Romero-Romero, Birte Höcker

**Affiliations:** a Department of Biochemistry, University of Bayreuth, 95447 Bayreuth, Germany; Institut Laue-Langevin, Grenoble, France

**Keywords:** periplasmic binding proteins, ribose binding protein, *Thermotoga maritima*, flavodoxin-like fold, circular permutation, domain swapping, protein evolution

## Abstract

Periplasmic binding proteins (PBPs) are a class of proteins that participate in the cellular transport of various ligands. They have been used as model systems to study mechanisms in protein evolution, such as duplication, recombination and domain swapping. It has been suggested that PBPs evolved from precursors half their size. Here, the crystal structures of two permuted halves of a modern ribose-binding protein (RBP) from *Thermotoga maritima* are reported. The overexpressed proteins are well folded and show a monomer–dimer equilibrium in solution. Their crystal structures show partially noncanonical PBP-like fold type I conformations with structural deviations from modern RBPs. One of the half variants forms a dimer via segment swapping, suggesting a high degree of malleability. The structural findings on these permuted halves support the evolutionary hypothesis that PBPs arose via a duplication event of a flavodoxin-like protein and further support a domain-swapping step that might have occurred during the evolution of the PBP-like fold, a process that is necessary to generate the characteristic motion of PBPs essential to perform their functions.

## Abstract 2

The crystal structures of two permuted halves of a ribose-binding protein from *Thermotoga maritima* provide insights into the evolution of the periplasmic binding protein fold from a flavodoxin-like precursor by duplication and domain swapping.

## 1. Introduction

Understanding the emergence of modern protein structures can be addressed by investigating the mechanisms that evolution might have employed. Some of the drivers for structural diversification are genetic mechanisms, such as mutation, duplication and recombination of domain-sized or even subdomain-sized protein fragments, offering the structural complexity needed for functions to evolve (Romero-Romero *et al.*, 2021 ▸; Sikosek & Chan, 2014 ▸; Höcker, 2014 ▸; Ohta, 2000 ▸). Another mechanism expanding this repertoire is domain swapping. While domain swapping does not lead to a change in protein sequence, its influence on the structure by forming oligomers via exchange of structural elements within the topology of a protein also contributes to the emergence of functions (Bennett *et al.*, 1995 ▸). Insights into these characteristics can shed light not only on the evolutionary history of proteins but also on our understanding of the determinants of protein folding in general.

One group of proteins that have been used for this purpose are periplasmic binding proteins (PBPs). They are involved in the cellular transport of a wide variety of small molecules such as carbohydrates, amino acids, vitamins and ions (Chandra­vanshi *et al.*, 2021 ▸; Felder *et al.*, 1999 ▸). The structurally symmetric bilobal architecture of their fold has long been thought to originate from a duplication and fusion event of an individual lobe (Fukami-Kobayashi *et al.*, 1999 ▸; Louie, 1993 ▸). While more detailed classifications of their fold exist (Scheepers *et al.*, 2016 ▸), they can be structurally separated into PBP-like fold types I and II, with somewhat different arrangements of secondary-structure elements. It has been proposed that type II PBPs derive from a tandem domain swap of type I PBPs, leading to exchange of the (βα)_5_ elements between the lobes (Fukami-Kobayashi *et al.*, 1999 ▸). Similar domain dislocation has previously been described in related protein folds such as the chemotaxis response regulator CheY (Paithankar *et al.*, 2019 ▸), the receiver domain of cytokinin receptor CRE1 (Tran *et al.*, 2021 ▸), the tryptophan synthase subunit TrpA (Michalska *et al.*, 2020 ▸) and the uroporphyrinogen III synthase (Toledo-Patiño *et al.*, 2019 ▸; Szilágyi *et al.*, 2017 ▸).

**Figure 1:**
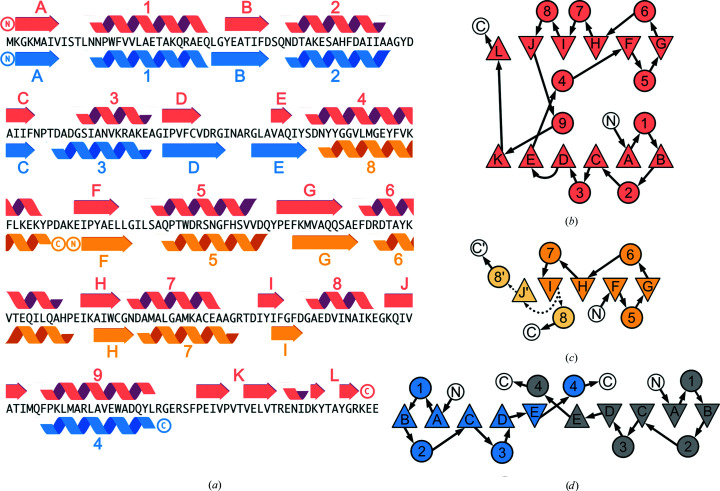
Secondary structure and topology of RBP and its permuted halves. (*a*) Secondary-structure alignment with the amino-acid sequence of RBP. Secondary-structure annotations derived from *PDBsum* (Laskowski *et al.*, 2018 ▸) are colored salmon for RBP, blue for RBP-CP_N_ and yellow for RBP-CP_C_. β-Sheets are sequentially labeled with letters in the order of the sequence and α-helices are labeled with numbers. These labels correspond to the topology representation (*b*, *c*, *d*) adapted from Fukami-Kobayashi *et al.* (1999 ▸), where β-sheets are depicted as triangles and α-helices as circles. The arrangement of the secondary-structure elements reflects their three-dimensional order for RBP (*b*), RBP-CP_C_ (*c*) and RBP-CP_N_ (*d*). The N- and C-termini are labeled N and C, respectively, and the connections between the secondary-structure elements are shown as arrows. The connections of the two possible configurations of β-strand I, either to α-helix 8 or β-strand J′, in RBP-CP_C_ are shown as dotted arrows as these stretches are not resolved in the crystal structure.

To investigate the structural flexibility of the α/β architecture found in type I PBPs, we separated and investigated the individual lobes of the ribose-binding protein from *Thermotoga maritima* (RBP; Cuneo *et al.*, 2008 ▸). An established way to stabilize and isolate structural units within a given protein fold is the use of circular permutations (Huang, Nayak *et al.*, 2011 ▸; Iwakura *et al.*, 2000 ▸; Hennecke *et al.*, 1999 ▸). Following this approach, two protein variants that structurally represent each lobe of RBP were created and characterized (Fig. 1 ▸). We successfully obtained crystal structures of both the N-terminal lobe (RBP-CP_N_) and the C-terminal lobe (RBP-CP_C_), observing a non-native swapping of elements in RBP-CP_N_. Our experiments also indicate dimerization of this lobe in solution, with the crystal structure showing a rearrangement reminiscent of the antiparallel β-sheet observed in type II PBPs. The observed structural malleability and the propensity to rearrange secondary-structural elements furthermore suggest a possible mechanism for transition from the type I PBP-like fold to type II via domain dislocation.

## 2. Materials and methods

### 2.1. Construct designs with *Rosetta*

The *RosettaRemodel* protocol included in the *Rosetta* suite (release 2018.19; Huang, Ban *et al.*, 2011 ▸) was used to sample possible loop conformations to connect the secondary-structure elements of the RBP lobes, leading to both the RBP-CP_N_ and RBP-CP_C_ sequences. The unliganded structure of *T. maritima* RBP (PDB entry 2fn9; Cuneo *et al.*, 2008 ▸), trimmed to include only the residues of the respective lobe, was used as a template. The new termini for the permuted constructs were introduced at positions 1 and 263 for RBP-CP_N_, with a loop inserted between positions 105 and 244 (strand E and helix 9; Fig. 1 ▸ *a*). For RBP-CP_C_ the N-terminus was shifted to residue 128, and a loop was inserted to connect residue 243 to the new C-terminal stretch from 106 to 127 (strand D and helix 4; Fig. 1 ▸ *a*). Flexibility of the input model was allowed for one additional residue on each side of the gap during loop closure. 1000 models of three- and four-residue loops were generated using parallelized processing with Open MPI and procedural seed generation. The top ten scoring models were relaxed using the *relax* algorithm provided in this version of *Rosetta*, and the total and per-residue scoring functions were used. The sequences of the best scoring models for both RBP-CP_N_ and RBP-CP_C_ were used as final constructs (Table 1 ▸). The per-residue energies of the relaxed models were compared with the unrelaxed crystal structure of RBP and the obtained crystal structures of RBP-CP_N_ and RBP-CP_C_ using the *score_jd*2 application in the same version of *Rosetta*.

### 2.2. Cloning and protein purification

The gene fragments for full-length RBP as well as RBP-CP_C_ were subcloned into empty linearized pET-21b(+) using NdeI/XhoI restriction sites. To prevent translation of the truncated sequence in wild-type RBP, an M142A mutation (Cuneo *et al.*, 2008 ▸) was introduced via QuikChange site-directed mutagenesis. The resulting plasmids were verified by sequencing. Gene synthesis and cloning into pET-21b(+) for RBP-CP_N_ were provided by Biocat. Transformant *Escherichia coli* BL21 (DE3) cells were grown in Terrific broth medium (TB) at 37°C to an OD_600_ of 1.2 in the presence of 100 µg ml^−1^ ampicillin. Protein expression was induced by the addition of 1 m*M* isopropyl β-d-1-thiogalactopyranoside and continued for 18 h at 20°C. The cells were harvested by centrifugation (5000*g*, 15 min), resuspended and lysed by sonication. To remove cell debris, the suspension was centrifuged again (40 000*g*, 1 h) and the supernatant was filtered through a 0.22 µm filter prior to immobilized metal ion chromatography (IMAC).

IMAC was performed on a Cytiva HisTrap 5 ml column previously equilibrated with buffer (20 m*M* MOPS, 500 m*M* NaCl, 10 m*M* imidazole pH 7.8). Elution was performed with a 40% step of elution buffer (20 m*M* MOPS, 500 m*M* NaCl, 600 m*M* imidazole pH 7.8). Fractions containing the protein of interest were pooled and concentrated for the size-exclusion chromatography step. Size-exclusion chromatography was performed on a Cytiva Superdex 26/600 75 pg column with isocratic elution of buffer (20 m*M* Tris–HCl, 300 m*M* NaCl pH 7.8). Fractions containing protein were analyzed by SDS–PAGE and those containing the proteins of interest were pooled, flash-frozen in liquid nitrogen and stored at −20°C until further analysis.

### 2.3. Crystallization

Initial crystallization screens were set up using a Phoenix pipetting robot (Art Robbins Instruments) with commercially available sparse-matrix screens (Qiagen; JCSG Core I–IV Suites and The PEGs Suite and PEGs Suite II) in 96-well sitting-drop plates (3-drop Intelli-Plates, Art Robbins Instruments). Droplets were pipetted in 1:1, 1:2 and 2:1 ratios of protein:reservoir solution with a protein concentration of 30 mg ml^−1^ and were incubated at 293 K. Initial crystals of RBP-CP_N_ appeared after 35 days in the following condition: 30% PEG 4000, 0.2 *M* lithium sulfate, 0.1 *M* Tris–HCl pH 8.5 (JCSG Core IV Suite) in the 1:1 ratio droplet. Subsequent optimization with Additive Screen (Hampton Research) yielded well diffracting cuboid-shaped crystals in the presence of the abovementioned initial hit solution supplemented with 4% 2,2,2-trifluoroethanol. Further cryoprotection was not needed.

RBP-CP_C_ was crystallized in the same fashion with a protein concentration of 15 mg ml^−1^. Diffracting cuboid-shaped crystals were found after one month in 0.2 *M* magnesium acetate, 20% PEG 3350 (The PEGs Suite) in the 1:2 ratio droplet. Cryoprotection was ensured by transferring the crystal to 20% PEG 3000, 20% ethylene glycol, 0.2 *M* KNO_3_.

### 2.4. X-ray data collection, structure determination and model building

Crystals were manually mounted using cryo-loops on SPINE standard bases and were flash-cooled after cryoprotection if needed. Diffraction data were collected on BL14.1 at the BESSY II electron-storage ring operated by the Helmholtz-Zentrum Berlin (Mueller *et al.*, 2015 ▸). Measurements were performed at 100 K in single-wavelength mode at 0.9184 Å with a Dectris PILATUS 6M detector in fine-slicing mode (0.1° wedges) using the *MXCuBE* beamline-control software (Gabadinho *et al.*, 2010 ▸). Data were processed with *XDSAPP*2 (Sparta *et al.*, 2016 ▸) employing *XDS* (Kabsch, 2010 ▸). Data quality was assessed by applying *phenix.xtriage* (Zwart *et al.*, 2005 ▸). Resolution cutoffs were determined by applying the automated paired refinement protocol *PAIREF* (Malý *et al.*, 2020 ▸).

In both cases, phases were solved by molecular replacement using the respective lobe of RBP (PDB entry 2fn9) as a search model with *Phaser* (McCoy *et al.*, 2007 ▸). The resulting models were manually rebuilt with *Coot* (Emsley *et al.*, 2010 ▸) and refined with *phenix.refine* (Afonine *et al.*, 2018 ▸) in an iterative manner. Coordinates and structure factors were validated and deposited in the PDB (Berman *et al.*, 2002 ▸) with accession codes 7qsq (RBP-CP_N_) and 7qsp (RBP-CP_C_).

### 2.5. Far-UV circular dichroism

Far-UV circular dichroism (CD) was measured on a Jasco J-710 spectropolarimeter equipped with a Peltier device (PTC-348 WI) to control the temperature at 20°C. Before the measurements, the protein samples were dialyzed overnight into 10 m*M* sodium phosphate pH 7.8, 50 m*M* sodium chloride. Samples were measured at a protein concentration of 10 µ*M* in a 2 mm cuvette in a wavelength range from 195 to 260 nm with a bandwidth of 1 nm. After subtraction of the buffer signal, the measured ellipticity signal was converted to mean residue molar ellipticity ([Θ]) using [Θ] = Θ/(*lCN*_r_), where Θ is the ellipticity signal in millidegrees, *l* is the cell path in millimetres, *C* is the molar protein concentration and *N*_r_ is the number of amino acids per protein (Greenfield, 2006 ▸).

### 2.6. Intrinsic fluorescence

Intrinsic fluorescence (IF) spectra were collected on a Jasco FP-6500 spectrofluorometer. Measurements were performed at 20°C controlled with a water bath (Julabo MB). Samples were dialyzed and the concentration was set as described previously for CD measurements. The excitation wavelength was set to 280 nm and emission was measured in the range 300–500 nm with a bandwidth of 1 nm. The raw signal was corrected for protein concentration and further normalized to relative fluorescence.

### 2.7. Size-exclusion chromatography–multi-angle light scattering

Size-exclusion chromatography–multi-angle light scattering (SEC-MALS) measurements were performed with a miniDAWN detector and an Optilab refractometer (Wyatt Technology) coupled to an analytical size-exclusion chromatography column (Superdex 75 Increase 10/300 GL). Centrifuged samples were run on the column connected to an ÄKTApure FPLC system (GE Healthcare Life Sciences) and equilibrated with 10 m*M* sodium phosphate pH 7.8, 50 m*M* sodium chloride, 0.02% sodium azide at room temperature. Measurements were run at a constant flow rate of 0.8 ml min^−1^ at protein concentrations of 0.5, 1.0 and 5 mg ml^−1^. The system setup was normalized and checked by measurement of a commercially available standardized BSA sample (2 mg ml^−1^; Pierce, catalogue No. 23209) before and after each series of measurements. Weight-averaged molar-mass determination was performed using the Zimm equation with the differential refractive-index signal as a source for the concentration calculations (the refractive-index increment d*n*/d*c* was set to 0.185). Analysis of the experiments was performed using the *ASTRA* version 7.3.2 software suite (Wyatt Technology).

### 2.8. Differential scanning calorimetry

Differential scanning calorimetry (DSC) endotherms were collected using a MicroCal PEAQ-DSC instrument (Malvern Panalytical) with protein concentrations of 0.5, 1.0 and 5 mg ml^−1^, a temperature range of 10–130°C and a scan rate of 1.5°C min^−1^. All samples were prepared after exhaustive dialysis in 10 m*M* sodium phosphate pH 7.8, 50 m*M* sodium chloride. After proper instrument equilibration with at least two buffer–buffer scans, physical and chemical baselines were subtracted from protein–buffer scans and the data were normalized by protein concentration. *Origin* version 9.0 (OriginLab Corporation) was used for data analysis.

## 3. Results and discussion

### 3.1. Design of RBP-CP_N_ and RBP-CP_C_

To assess how the individual lobes of a PBP-like fold behave, we chose the ribose-binding protein from *T. maritima* (RBP). Due to its thermophilic nature, it was considered to be a robust model system that could more readily tolerate this manipulation. In addition, it has previously been reported that this protein is expressed as a 21 kDa truncation (Cuneo *et al.*, 2008 ▸), suggesting that at least some elements of this protein may exist in isolation. To isolate the two lobes of RBP, the elements that make up the individual two halves were linked together via an artificial loop (Table 1 ▸). The resulting constructs RBP-CP_N_ (N-terminal lobe) and RBP-CP_C_ (C-terminal lobe) represent the two symmetric lobes of the PBP-like fold (Figs. 1 ▸ *a* and 1 ▸ *b*). The specific intersections were determined by structural alignment of the crystal structure of RBP from *T. maritima* in the absence of its ligand ribose (PDB entry 2fn9). RBP-CP_N_ was designed to consist only of the β_A–E_α_1–4_ elements, which are directly linked to α_9_. Similarly, RBP-CP_C_ consists of the elements β_F–J_α_5–8_ connected to α_4_ of RBP by permutation (Fig. 1 ▸ *a*). To be consistent with the structure of the theoretical evolutionary precursor before duplication, the additional secondary-structural elements at the C-terminus of RBP (β_K–L_) responsible for the second crossover between the two lobes were removed.

**Figure 2:**
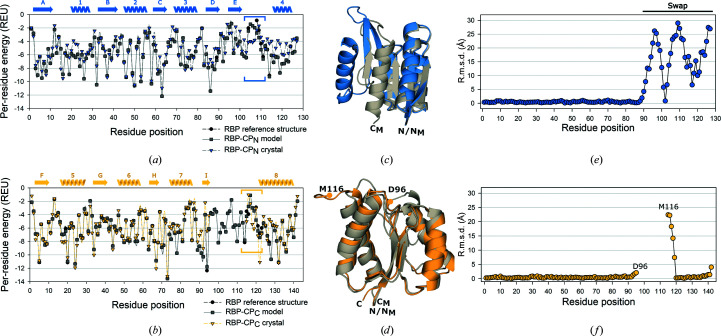
Per-residue *Rosetta* energy terms and comparison of the per-residue r.m.s.d. of the models to the crystal structure. (*a*, *b*) Energies in Rosetta energy units (REU) for each residue position of the template RBP structure (black, circles, dashed line), the model of RBP-CP_N_ or RBP-CP_C_ (gray, squares, solid line) and the respective crystal structures (blue for RBP-CP_N_ and yellow for RBP-CP_C_, triangles, dashed lines). Sites where loop residues were introduced are highlighted by colored brackets for each protein. Secondary-structural elements as observed in the crystal are shown and are labeled as in Fig. 1 ▸(*a*). (*c*, *d*) Superposition of the computational models (gray) and the corresponding crystal structures of RBP-CP_N_ (blue) and RBP-CP_C_ (yellow). The borders of the area of missing density in RBP-CP_C_ are labeled D96 and M116. (*e*, *f*) Per-residue r.m.s.d. (based on C^α^ atoms) of the obtained crystal structures of RBP-CP_N_ (blue) and RBP-CP_C_ (yellow) compared with their models. The representation and alignment were obtained using *PyMOL* 2.5.0 (Schrödinger) and the align command with cycles=0, considering only C^α^ atoms of chains *A* and transferring per-residue values with the rmsd_b script (http://pldserver1.biochem.queensu.ca/~rlc/work/pymol/rmsd_b.py).

We obtained computational models of each lobe with comparable total and per-residue energies to the trimmed input structures of full-length RBP. Comparison of the scores obtained from the *Rosetta* energy function of native RBP and the models show similar energies for all structures (Figs. 2 ▸ *a* and 2 ▸ *b*). The similarity of the per-residue energy of RBP to the corresponding values for the models indicates that at least energetically, the added loop residues are suitable. The per-residue energies further show a similar distribution. For most of the sequence of RBP-CP_N_, the residue energies of the crystal structure are comparable to those of the model. Only the residues of the inserted loop (blue bracket in Fig. 2 ▸ *a*) score lower in the crystal structure compared to the computational model. However, the entire stretch after the inserted residues displays a higher energy (in Rosetta energy units; REU) than in the model. This is similarly reflected in both the structural rearrangement of the secondary-structure elements (Figs. 1 ▸ *d* and 2 ▸ *c*) and the per-residue r.m.s.d. in RBP-CP_N_ (Fig. 2 ▸ *e*). The observation is consistent with the dimerization interface being facilitated via swapping of the α_4_ element and disruption of the expected conformation at the C-terminus. While the deviation in r.m.s.d. for RBP-CP_N_ would imply a disturbance of per-residue energies in the C-terminal stretch (Fig. 2 ▸ *e*), the segment swap seems to compensate for it in canonical topology.

In contrast, a comparison of the scores of the RBP-CP_C_ model and its resulting crystal structure shows similar energies for all resolved residues (Fig. 2 ▸ *b*). The per-residue energies of the designed loop are also comparable, even though their conformation in the crystal differs significantly from the model (yellow bracket in Fig. 2 ▸ *b*). Apart from the residues around the stretch of missing density (Asp96–Met116), the predicted structure corresponds well to the obtained crystal structure (Fig. 2 ▸ *d*) and the per-residue r.m.s.d. values also indicate good agreement (Fig. 2 ▸ *f*).

### 3.2. Both lobes are stable proteins with a tendency to form dimers

**Figure 3:**
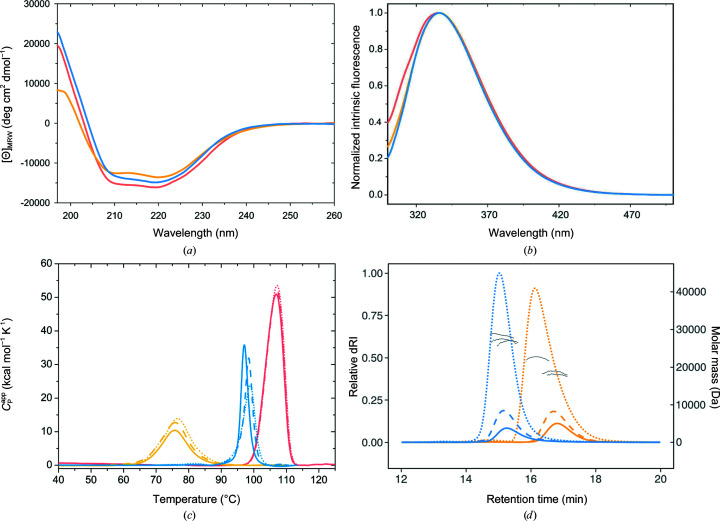
Biochemical characterization. (*a*) Far-UV CD spectra of RBP (salmon), RBP-CP_N_ (blue) and RBP-CP_C_ (yellow). (*b*) Normalized tryptophan fluorescence at a 280 nm excitation wavelength of RBP (salmon), RBP-CP_N_ (blue) and RBP-CP_C_ (yellow). (*c*) DSC endotherms of RBP (salmon), RBP-CP_N_ (blue) and RBP-CP_C_ (yellow); sample concentrations of 0.5, 1 and 5 mg ml^−1^ are shown as solid, dashed and dotted lines, respectively. (*d*) SEC-MALS analysis of RBP-CP_N_ (blue) and RBP-CP_C_ (yellow) at different concentrations. The elution profile is plotted as the relative differential refractive index against the retention time. Sample concentrations of 0.5, 1 and 5 mg ml^−1^ are shown as solid, dashed and dotted lines, respectively. Molar-mass determinations for peak regions are plotted as gray dots.

RBP-CP_N_ and RBP-CP_C_ could be expressed recombinantly in high yields in *E. coli* and purified to homogeneity. Far-UV CD spectra of both RBP-CP_N_ and RBP-CP_C_ show typical characteristics of a protein with an α/β-like structure and are comparable to that of full-length RBP (Fig. 3 ▸ *a*). In addition, an initial hint about the correct formation of the tertiary structure in solution was obtained from the intrinsic fluorescence spectra. The emission maximum at 335 nm for both proteins as well as for RBP indicates that the aromatic residues are in a hydrophobic core and are buried from solvent, confirming that all proteins adopt a comparable compact structure (Fig. 3 ▸ *b*). Another indication that the constructs appear to fold stably is the determination of thermal stability by differential scanning calorimetry (DSC). The DSC endotherms obtained for both RBP-CP_N_ and RBP-CP_C_ show a single and highly cooperative transition (Fig. 3 ▸ *c*). The thermal unfolding appears to be irreversible, as no transition is observed upon cooling and the measurement of a second heating cycle. The permuted constructs show a lower thermostability than full-length RBP, with *T*_m_ values of 76.1 ± 0.4°C for RBP-CP_C_ and 97.9 ± 0.9°C for RBP-CP_N_, in contrast to 108°C for RBP (Cuneo *et al.*, 2008 ▸). There also appears to be a small dependence on protein concentration, with a shift to higher transition temperatures at higher protein concentrations (Fig. 3 ▸ *c*).

Since the architecture of PBPs is likely to have originated from an ancestral dimer with the canonical binding site between the lobes, the question arises whether both variants can adopt a similar conformation. To investigate this, the oligomeric state of the proteins was determined in solution using SEC-MALS measurements (Fig. 3 ▸ *d*). In the concentration range 0.5–5 mg ml^−1^, the determined molecular weight (MW) of RBP-CP_N_ is approximately 27.5 kDa. This corresponds to a dimeric conformation, as it is about double the expected monomeric MW of 14.9 kDa. The shift from lower molecular weight at lower concentrations to higher molecular weight at higher concentrations indicates that the monomer–dimer equilibrium is dynamic and concentration-dependent. A similar pattern is observed for RBP-CP_C_. While the protein appears to be monomeric at low concentrations (0.5 mg ml^−1^), the MW shifts to 18.7 kDa at 1 mg ml^−1^ and to 22.4 kDa at 5 mg ml^−1^. This would correspond to a dynamic shift from a monomer (theoretical MW of 16.7 kDa) to a dimer (Table 2 ▸). These results are in agreement with the concentration-dependent thermostability observed in DSC measurements. Together, they explain the shift to higher temperatures during thermal unfolding, with possible stabilization of the overall fold by forming a defined dimer interface.

### 3.3. The structures of both RBP-CP_N_ and RBP-CP_C_ differ from their native counterparts

The PBP-like type I canonical fold consists of two lobes with a continuous, parallel β-sheet with five strands in the order 21345 plus an additional, noncontinuous β_6_ strand flanked by alternating α-helices on each side and one crossover between each lobe (Figs. 1 ▸ *a* and 1 ▸ *b*). In contrast to the expected single-lobed architecture, the crystal structures obtained for RBP-CP_N_ and RBP-CP_C_ deviate from the structure of full-length RBP.

**Figure 4:**
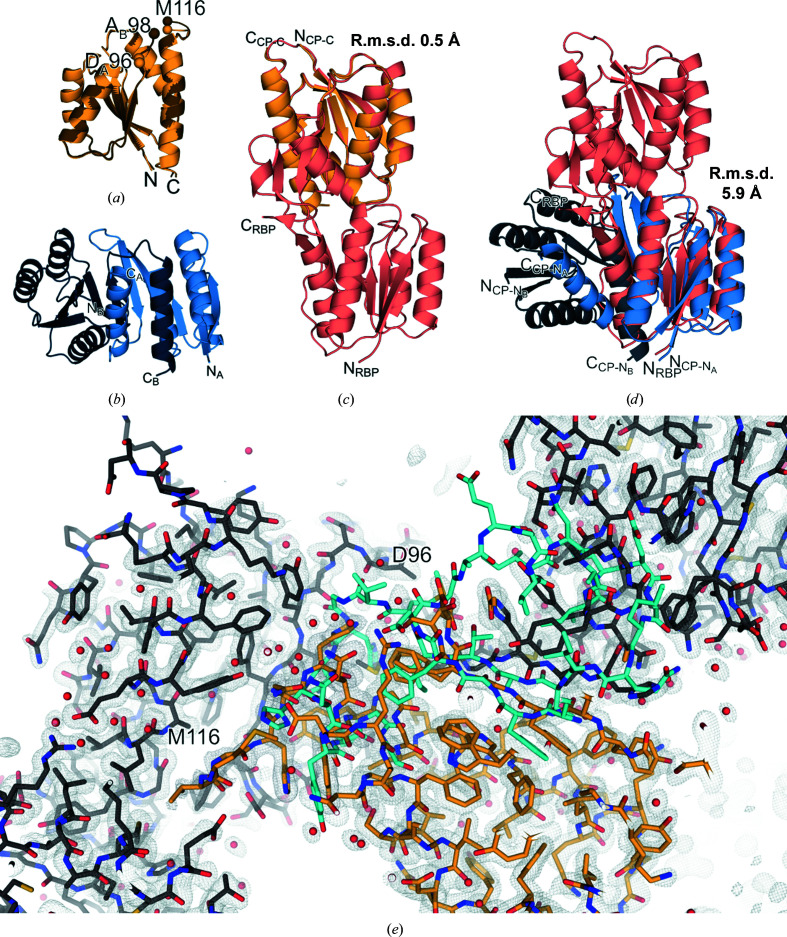
Comparison of the crystal structures of the individual lobes with full-length RBP. (*a*) Cartoon representation of the structural alignment of the two chains in the asymmetric unit of RBP-CP_C_, with the edges of the unresolved region of chains *A* (Asp95–Met116) and *B* (Ala98–Met116) shown as spheres. (*b*) Cartoon representation of the crystallographic dimer of RBP-CP_N_. (*c*, *d*) Superposition of the cartoon structures of full-length RBP with RBP-CP_C_ (*c*) and RBP-CP_N_ (*d*), respectively. R.m.s.d. values over all C^α^ atoms of chain *A* of each structure are provided next to each figure. (*e*) Missing density in the RBP-CP_C_ map spanning residues Asp96–Met116. The crystal structure is shown as sticks, where chain *A* is colored yellow and symmetry mates are colored gray. A stick representation of the corresponding *Rosetta* model (residues Ile92–Gly125) is shown as an overlay in cyan. Water molecules are depicted as red spheres. A 2*F*_o_ − *F*_c_ map contoured at an r.m.s.d. of 1.0 is shown as gray mesh. The representation and alignment were obtained using *PyMOL* 2.3.0 (Schrödinger) and the align command with cycles=0.

**Figure 5:**
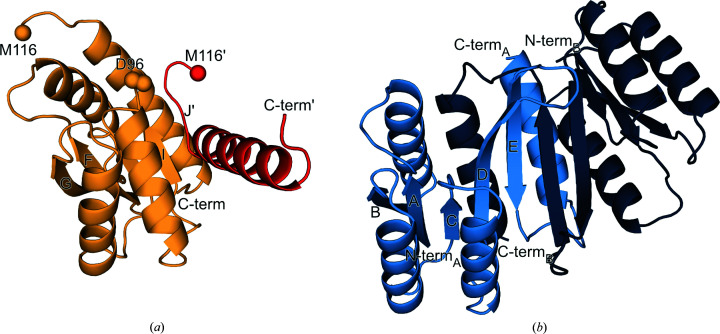
Possible alternative interface facilitated by a symmetry mate in the crystal structure of RBP-CP_C_. (*a*) Cartoon representation of the interface of chain *A* and the participating elements of its symmetry mate chain *A*′ in the crystal structure of RBP-CP_C_. The termini of the protein and the gap where the chain could not be traced are labeled for each chain. (*b*) Cartoon representation of the interface of the RBP-CP_N_ dimer. Secondary structures are labeled according to Fig. 1 ▸.

RBP-CP_C_ crystallized in the orthorhombic space group *P*2_1_2_1_2_1_, with two chains of the protein in the asymmetric unit, and was refined to a resolution of 1.36 Å (Table 3 ▸). While the N-terminal (αβ)_4_ elements in both chains are nearly identical to the core of the corresponding part in full-length RBP, the remaining elements differ from the canonical topology (Figs. 1 ▸ *b* and 1 ▸ *c*). While the core structure of α_5–7_ and β_F–I_ in RBP-CP_C_ is comparable to that of RBP, the following β_J_ strand and the synthetic loop are not resolved in the crystal structure (Fig. 4 ▸ *a*). However, the connecting α_8_ helix on the other side of this gap in the structure can unambiguously be seen (Fig. 1 ▸ *c*). It remains unclear whether the inserted loop or the energetical frustration of missing elements on this terminal side of the protein interferes with the proper formation of β_J_, or whether a preferential but unobserved swap of elements with an adjacent protein molecule results in the lack of density in this protein region (Fig. 4 ▸ *e*). An alternative explanation could be the formation of an interface between two crystallographic dimers, as indicated by an analysis with the *PISA* server (Krissinel & Henrick, 2007 ▸). In this case, the C-terminal α_8_ would not originate from the same chain of the asymmetric unit but from its corresponding symmetry mate. The resulting extended arrangement is facilitated by an interaction of the β_I_ strand and the residue stretch 116′–120′ (Fig. 5 ▸ *a*). This extension is similar to a continuation of the sheet via the antiparallel addition of a short, single stretch resembling a strand, with the residues of the designed loop (Val117–His121) participating in the interaction (Fig. 1 ▸ *c*). With the α_4_ helix originating from the adjacent symmetry mate, it is also possible that there is a mixed population of both conformations, with the helix serving as a common structural anchor point. This could also explain the lack of density in the connecting area. A similar shuffling of elements can be observed with less ambiguity in the crystal structure of RBP-CP_N_ (Fig. 5 ▸ *b*). This possible interaction could also explain the concentration-dependent oligomerization observed in the SEC-MALS measurements (Fig. 3 ▸ *d*). The central β-sheet as well as all α-helices appear to be well ordered, except for the loops close to the unresolved region and the termini. The r.m.s.d. of 0.5 Å over 135 C^α^ atoms of the resolved residues, however, indicates a high similarity between RBP-CP_C_ and the corresponding elements of full-length RBP (Fig. 4 ▸ *c*).

The case is different when looking at the N-terminal lobe. The crystal structure of RBP-CP_N_ was solved in the monoclinic space group *P*2_1_ at 1.79 Å resolution. The asymmetric unit is composed of four chains, of which two pairs form a dimer via a segment swap. Unlike the interface of the two lobes in native PBPs, the dimer is located on the edge of the two central β-sheets (Fig. 4 ▸ *b*). This extension of the sheet is mediated via each of the respective β_E_ strands. In contrast to the rest of the central β-sheet, the two β_E_ strands form an antiparallel stretch of the extended β-sheet. This change in direction of the C-terminal β-strand is not known to occur in PBP-like fold type I proteins, in which the central β-sheet always adopts a parallel conformation. In addition, this swap of the β_D_β_E_ elements in their parallel–antiparallel arrangement forms the interface of the dimer (Fig. 1 ▸ *d*). These structural rearrangements are also reflected by the significant difference in r.m.s.d. of 5.9 Å when comparing the structure of RBP-CP_N_ with the equivalent half of the full-length RBP (Fig. 4 ▸ *d*). This unusual rearrangement of elements indicates a high tolerance of this structural motif to variations in its topology. In agreement with other structures, such as the CheY-like fold (Paithankar *et al.*, 2019 ▸), the TIM-barrel fold (Michalska *et al.*, 2020 ▸) and other related folds (Lewis *et al.*, 2000 ▸; Tran *et al.*, 2021 ▸; Szilágyi *et al.*, 2017 ▸), the isolated domains of a PBP-like type I protein show a high degree of malleability.

## 4. Conclusions

The obtained crystal structures of the permuted constructs of both the N- and C-terminal lobes of RBP from *T. maritima* suggest the possibility that they could have existed in isolation of the full structural context. This corresponds to the idea that modern PBPs arose from a duplication event. Based on structural and sequence similarities, it has been proposed that this progenitor was an ancestral protein of the flavodoxin-like fold. The existence of the stable permuted halves clearly shows that the single lobe can exist on its own and can help inform on this evolutionary process.

However, the observed swapping of elements in RBP-CP_N_ could also correspond to another event in the evolution of PBPs. It has previously been concluded that the evolution of the PBP-like fold involved domain swapping of the C-terminal helices, a step that was necessary to generate the characteristic hinge-bending motion of PBPs, with subsequent fusion of this proposed ancestral dimer (Fukami-Kobayashi *et al.*, 1999 ▸). In addition, it has been proposed that the absence of the helix between β-strands D and E and helix 8 (Fig. 1 ▸ *b*) may have been a necessary step for the swapping event that led to PBPs with the type II fold. This partially explains why we observe a dimer with an unusual segment swap in RBP-CP_N_, which lacks this helix. However, it appears that RBP-CP_C_, which still contains this corresponding helix 8, does not reliably form a dimer. However, the alternative interface involving the chain from a symmetry mate could partially explain the behavior observed in SEC-MALS measurements. The dynamic shift to higher molecular weight species can only be observed at high protein concentrations. Interestingly, however, the antiparallel stretch of residues 117′–119′ in RBP-CP_C_ bears a resemblance to the continuation of the central β-sheet in RBP-CP_N_. The residues participating in the interaction with β4 are the additional residues introduced via the design. A reason for this could be the energetically frustrated surface of β4, which now lacks the corresponding β5 from RBP, that induces the switch of the designed loop into a more strand-like conformation to satisfy this hydrophobic surface.

Alternatively, a possible explanation may lie in the folding pathway of proteins with a flavodoxin-like fold. The folding mechanism of CheY, a well studied protein with a flavodoxin-like fold, suggests that there may be a universal subdomain intermediate in the folding pathway (Hills & Brooks, 2008 ▸). The N-terminal β_1–3_α_1–2_ elements appear to initially form a central triad followed by folding of the remaining elements. The permuted RBP lobes could follow a similar path. The corresponding elements could form a folded scaffold onto which the rest of the protein folds. This substructure potentially stabilizes the protein to a point where the C-terminal elements can still adapt a structured conformation but provide sufficient flexibility for the unusual rearrangement that we have found.

The novel antiparallel stretch of the dimer-swapped β-sheets has not been observed before in proteins with the type I PBP-like fold, and the existence of this swap highlights the flexibility of this structural element. Additionally, the alleviation of the energetically frustrated hydrophobic surface achieved via the alternative interface in the structure of RBP-CP_C_ could offer valuable insights into the mechanisms behind domain swapping in PBPs in general. More detailed sequence analysis and experiments would be required to obtain a clear picture of the transition from type I to type II PBPs. The malleability of this α/β architecture, which is also apparent in other folds (for example the Rossmann, flavodoxin and TIM-barrel-like folds), may be a reason for its frequent occurrence in modern proteins (Ferruz *et al.*, 2021 ▸).

## Supplementary Material

PDB reference: RBP-CP_C_, 7qsp

PDB reference: RBP-CP_N_, 7qsq

## Figures and Tables

**Table 1:** Sequences of full-length RBP and the permuted RBP halves The M142A mutation in RBP and the residues inserted based on *Rosetta* modeling in RBP-CP_N_ and RBP-CP_C_ are highlighted in bold.

Name	Sequence
RBP	MKGKMAIVISTLNNPWFVVLAETAKQRAEQLGYEATIFDSQNDTAKESAHFDAIIAAGYDAIIFNPTDADGSIANVKRAKEAGIPVFCVDRGINARGLAVAQIYSDNYYGGVLAGEYFVKFLKEKYPDAKEIPYAELLGILS**A**QPTWDRSNGFHSVVDQYPEFKMVAQQSAEFDRDTAYKVTEQILQAHPEIKAIWCGNDAMALGAMKACEAAGRTDIYIFGFDGAEDVINAIKEGKQIVATIMQFPKLMARLAVEWADQYLRGERSFPEIVPVTVELVTRENIDKYTAYGRKLEHHHHHH
RBP-CP_N_	MKGKMAIVISTLNNPWFVVLAETAKQRAEQLGYEATIFDSQNDTAKESAHFDAIIAAGYDAIIFNPTDADGSIANVKRAKEAGIPVFCVDRGINARGLAVAQIYSD**TST**QFPKLMARLAVEWADQYLRGGHHHHHH
RBP-CP_C_	MKEIPYAELLGILSAQPTWDRSNGFHSVVDQYPEFKMVAQQSAEFDRDTAYKVTEQILQAHPEIKAIWCGNDAMALGAMKACEAAGRTDIYIFGFDGAEDVINAIKEGKQIVATIM**VGHNH**NYYGGVLAGEYFVKFLKEKYPDGGHHHHHH

**Table 2:** Molecular-weight determination with SEC-MALS

Sample (concentration)	Expected MW (kDa)	Experimental MW (kDa)	Uncertainty (%)
RBP-CP_N_ (0.5 mg ml^−1^)	14.9	26.8	0.8
RBP-CP_N_ (1.0 mg ml^−1^)		27.2	0.5
RBP-CP_N_ (5.0 mg ml^−1^)		28.5	0.3
RBP-CP_C_ (0.5 mg ml^−1^)	16.7	18.0	1.0
RBP-CP_C_ (1.0 mg ml^−1^)		18.7	0.7
RBP-CP_C_ (5.0 mg ml^−1^)		22.4	0.4

**Table 3:** Crystallographic data and refinement statistics

	RBP-CP_N_	RBP-CP_C_
PDB code	7qsq	7qsp
Wavelength (Å)	0.9184	0.9184
Resolution range (Å)	48.96–1.79 (1.86–1.79)	39.76–1.36 (1.40–1.36)
Space group	*P*2_1_	*P*2_1_2_1_2_1_
*a*, *b*, *c* (Å)	55.37, 62.77, 76.26	41.69, 41.97, 132.20
α, β, γ (°)	90, 102.1, 90	90, 90, 90
Total reflections	176181 (15604)	533879 (48154)
Unique reflections	47556 (4346)	50883 (4875)
Multiplicity	3.7 (3.6)	10.5 (9.9)
Completeness (%)	97.8 (85.5)	99.0 (96.7)
Mean *I*/σ(*I*)	8.58 (0.76)	13.93 (1.00)
Wilson *B* factor (Å^2^)	32.6	18.8
No. of molecules in asymmetric unit	4	2
Matthews coefficient (Å^3^ Da^−1^)	2.14	1.72
*R*_merge_	0.080 (1.324)	0.081 (1.907)
*R*_meas_	0.094 (1.548)	0.085 (2.008)
*R*_p.i.m._	0.047 (0.788)	0.026 (0.616)
CC_1/2_	0.997 (0.413)	0.999 (0.322)
CC*	0.999 (0.765)	1.000 (0.698)
Reflections used in refinement	47294 (4102)	50883 (4875)
Reflections used for *R*_free_	2088 (181)	2100 (201)
*R*_work_	0.191 (0.370)	0.171 (0.353)
*R*_free_	0.239 (0.396)	0.210 (0.380)
CC_work_	0.963 (0.685)	0.962 (0.605)
CC_free_	0.952 (0.532)	0.938 (0.554)
No. of non-H atoms		
Total	4446	2308
Macromolecules	4063	2073
Solvent	315	199
No. of protein residues	510	248
R.m.s.d., bond lengths (Å)	0.003	0.012
R.m.s.d., bond angles (°)	0.57	1.23
Ramachandran favored (%)	98.4	99.2
Ramachandran allowed (%)	1.4	0.8
Ramachandran outliers (%)	0.2	0.0
Rotamer outliers (%)	1.4	1.4
Clashscore	5.16	4.82
Average *B* factor (Å^2^)		
Overall	40.0	25.9
Macromolecules	39.2	24.6
Solvent	46.5	35.9
No. of TLS groups	4	2
